# Ras Inhibition Induces Insulin Sensitivity and Glucose Uptake

**DOI:** 10.1371/journal.pone.0021712

**Published:** 2011-06-29

**Authors:** Adi Mor, Elizabeta Aizman, Jacob George, Yoel Kloog

**Affiliations:** 1 Department of Neurobiology, George S. Wise Faculty of Life Sciences, Tel Aviv University, Tel-Aviv, Israel; 2 Department of Cardiology, Kaplan Medical Center, Rehovot, affiliated to the Hebrew University—Hadassah Medical School, Jerusalem, Israel; City of Hope National Medical Center and Beckman Research Institute, United States of America

## Abstract

**Background:**

Reduced glucose uptake due to insulin resistance is a pivotal mechanism in the pathogenesis of type 2 diabetes. It is also associated with increased inflammation. Ras inhibition downregulates inflammation in various experimental models. The aim of this study was to examine the effect of Ras inhibition on insulin sensitivity and glucose uptake, as well as its influence on type 2 diabetes development.

**Methods and Findings:**

The effect of Ras inhibition on glucose uptake was examined both *in vitro* and *in vivo*. Ras was inhibited in cells transfected with a dominant-negative form of Ras or by 5-fluoro-farnesylthiosalicylic acid (F-FTS), a small-molecule Ras inhibitor. The involvement of IκB and NF-κB in Ras-inhibited glucose uptake was investigated by immunoblotting. High fat (HF)-induced diabetic mice were treated with F-FTS to test the effect of Ras inhibition on induction of hyperglycemia. Each of the Ras-inhibitory modes resulted in increased glucose uptake, whether in insulin-resistant C2C12 myotubes *in vitro* or in HF-induced diabetic mice *in vivo*. Ras inhibition also caused increased IκB expression accompanied by decreased expression of NF-κB . In fat-induced diabetic mice treated daily with F-FTS, both the incidence of hyperglycemia and the levels of serum insulin were significantly decreased.

**Conclusions:**

Inhibition of Ras apparently induces a state of heightened insulin sensitization both *in vitro* and *in vivo*. Ras inhibition should therefore be considered as an approach worth testing for the treatment of type 2 diabetes.

## Introduction

Insulin resistance is defined as impaired sensitivity to insulin in its main target organs (muscle, liver and adipose tissues), and is considered a hallmark of type 2 diabetes [1]. Insulin levels regulate glucose uptake by a variety of mechanisms, including induction of glucose transporter 4 (Glut4) expression, enhancement of translocation of the transporter to the muscle tissue membranes, reduction of free fatty acid (FFA) secretion from adipocytes, and inhibition of gluconeogenesis in the liver. Resistance to insulin results in increased concentrations of circulating FFA, which inhibits glucose uptake by muscle cells and increases glucose production by the liver [2].

Recent findings point to interrelationships between inflammation, insulin resistance, and type 2 diabetes. Lipid accumulation in the adipose tissue and expansion of the fat mass can initiate an inflammatory process, accompanied by local production and secretion of pro-inflammatory cytokines and chemokines [3], [4]. Pro-inflammatory cytokines such as tumor necrosis factor-α (TNF-α) reduce insulin sensitivity in muscle tissue and stimulate hepatic lipogenesis and hyperlipidemia [5], [6], [7]. Hepatic steatosis promotes low-grade inflammation via activation of nuclear factor-κB (NF-κB) [8]. The affected adipose, muscle and liver tissues together create an inflammatory milieu that promotes insulin resistance locally [9].

In an insulin-resistant state, serine kinases phosphorylate insulin receptor substrate (IRS), which results in inhibition of insulin signaling. A prominent participant in this process is the inhibitor of κB kinase (IKK), which phosphorylates, among other molecules, the insulin receptor. It also phosphorylates IκB, inducing the release of nuclear factor-κB (NF-κB) from IκB and allowing it to enter the nucleus [10]. NF-κB promotes upregulation of mediators that enhance inflammation and induce disease progression [11], [12], [13].

A prominent protein family that participates in the regulation of intracellular signal transduction and exerts a major impact on inflammation is the family of Ras GTPases [14], [15]. These small (∼21 kDa) proteins consist of molecular switches that regulate cell growth, differentiation, survival, migration and death [15], [16], [17], [18], [19], [20], [21], [22]. Ras is crucially involved in the proper activity of many cell types, including immune cells. Therefore, its abnormal involvement in cancer and autoimmune diseases has been the subject of intensive research [23], [24], [25], [26], with many studies aimed at understanding the possible involvement of Ras signaling in the disease and at developing selective inhibition of the active Ras protein. A well characterized protein activated by Ras is the AKT protein. Through stimulation of PI3K, Akt/PKB kinase is activated and phosphorylates the IKK, which in turn activates NF-κB. Inhibition of Ras can therefore attenuate NF-κB activation and reduce the inflammatory process [27].

S-trans,trans-farnesylthiosalicylic acid (FTS, Salirasib) is a small synthetic molecule that acts as a potent Ras inhibitor by competing with the anchoring of active Ras to the plasma membrane. Our group has described a number of FTS analogs that also act as Ras inhibitors, the most potent being 5-fluoro-FTS (F-FTS) [28]. The effect of FTS and its analogs was studied in various animal models of immune-mediated experimental disorders and found to significantly attenuate disease progression [29]. The attenuation was accompanied by altered gene expression in Ras signaling pathways, including the NF-κB signaling cascades [23], [24], [25], [26], [30], [31], [32].

In the present study we attempted to gain a better understanding of the effects of Ras inhibition on insulin resistance and type 2 diabetes by treating differentiated myotubes *in vitro* and high-fat (HF)-induced diabetic mice *in vivo* with DN-Ras or the synthetic Ras inhibitor F-FTS. We examined the effects of such treatment on glucose uptake and cellular signaling pathways, with particular focus on NF-κB-dependent signaling cascades. We found that treatment with the Ras inhibitor, F-FTS, reduced insulin-resistance *in vitro* and attenuated type 2 diabetes *in vivo.* The effects of Ras inhibition were mediated by the IKB/NF-κB cascade.

## Materials and Methods

### Induction of insulin resistance in cell culture

Mouse C2C12 myoblasts (generously provided by Prof. David Yaffe) were maintained in DMEM supplemented with 10% fetal bovine serum (FBS), 50 U/ml penicillin, and 50 µg/ml streptomycin. When cells reached confluence, the medium was replaced by differentiation medium containing DMEM and 2% horse serum, which was changed every other day. After 4 more days the differentiated C2C12 cells had fused into myotubes. To induce insulin resistance in the differentiated skeletal muscle cells, the medium was replaced by lipid-containing medium. The latter was prepared by addition of FFA (palmitate dissolved in 0.1 M NaOH) to DMEM containing 2% fatty acid-free BSA. Myotubes were incubated for 16 h in the above medium in the presence or absence of 0.75 mM palmitate. To exclude the possibility that any FFA can induce insulin resistance, 0.75 mM oleic acid was also added to myotubes and served as negative control (data not shown).

### Determination of glucose uptake by differentiated C2C12 skeletal muscle cells

Following induction of insulin resistance, all culture medium was removed from each well and replaced with 1 ml of fresh culture medium in the absence or presence of 10 µM fluorescent 2-NBDG (Molecular Probes-Invitrogen, CA/Molecular Probes, Eugene, OR), a new fluorescent derivative of glucose with a 2-[*N*-(7-nitrobenz-2-oxa-1,3-diazol-4-yl)] amino group at the C-2 position [33]. For this purpose, the cells were incubated at 37°C with 5% CO_2_ for 1 h. The cells were then washed twice with cold phosphate-buffered saline (PBS) and collected for flow cytometric measurement.

### Transfection with dominant-negative Ras

To block Ras we transfected differentiated C2C12 cells with 2 7g of green fluorescent protein plasmid (pGFP) or dominant-negative (DN) GFP-Ras (17N), using lipofectamine 2000 reagent according to manufacture's instructions (Invitrogen, Carlsbad, CA, USA). At 48 h post-transfection the cells were subjected to induction of insulin resistance, as described above. The cells were then either harvested and analyzed by Western blotting or tested for glucose uptake using the 2-NBDG method described above.

### Western blotting

To examine the impact of Ras inhibition on IκB and NF-κB, we performed Western immunoblotting with specific antibodies. Muscle and fat lysates were obtained from HF-induced mice, subjected to sodium dodecyl sulfate–polyacrylamide gel electrophoresis (SDS-PAGE), and Western blotted as previously described [30] with one of the following antibodies: anti-IκB, anti-p-IκB, anti-NF-κB (Santa Cruz Biotechnology, Santa Cruz, CA) or anti-tubulin (eBioScience, San Diego, CA).

The levels of Ras GTP were determined as described previously [30]. Protein bands were visualized with an enhanced chemiluminescence kit (Amersham Pharmacia Biotech, Arlington Heights, IL) and quantified by densitometry with Image EZQuant-Gel software©.

### Glut4 expression determined by reverse transcription–PCR

RNA was extracted from 10^6^ C2C12 muscle cells using an RNeasy Mini Kit (Qiagen, Hilden, Germany) according to the manufacturer's instructions. Reverse transcription (RT)–PCR was performed according to the protocol of the Reverse-iT™ 1^st^ Strand Synthesis Kit (ABgene, Epsom, UK). Glyceraldehyde-3-phosphate dehydrogenase (GAPDH) was analyzed using the following primers: GAPDH forward 5′-ACCACAGTCCATGCCATCAC-3′ and GAPDH reverse 5′-TCCACCACCCTGTTGCTGTA-3′.

PCR was carried out with ReddyMix™ PCR Master Mix (ABgene) on a Programmable Thermal Controller (MJ Research, Waltham, MA) at gene-specific conditions. Primer sequences for Glut4 were: Glut4 forward: 5′-GATGCCGTCGGGTTTCCAGCA-3′ and Glut4 reverse: 5′-TGAGGGTGCCTTGTGGGATGG -3′.

The PCR products were subjected to electrophoresis in 2% agarose gel stained with ethidium bromide.

### *In vivo* studies

The study was approved by the Institutional Ethics Committee at the Tel Aviv Sourasky Medical Center, Tel Aviv, Israel (Approval ID is L-09-006).

### Hydrodynamic delivery and determination of glucose uptake *in vivo*

C57Bl/6 mice *(n* = 8) received hydrodynamic DNA injections, in which 100 µg of pDN-Ras-GFP in 2 ml of PBS was injected intravenously (i.v.) and rapidly (within 5–8 s) under high-pressure into the tail vein. After 48 h the mice were injected i.v with 500 µg of 2-NBDG. Two hours later, muscle, liver and fat tissues were removed and single-cell suspensions from each of those tissues were tested by fluorescence activated cell sorting (FACS) for the presence of 2-NBDG. Mice in a control group (*n* = 8) were similarly injected and treated, except that they were injected with pGFP instead of pDN-Ras-GFP.

We produced a model for type 2 diabetes by feeding a high-fat diet (TD.06415, Harlan Laboratories, Madison, WI, USA) to male C57Bl/6 mice, starting when they were 6 weeks old as detailed earlier [34]. These high fat-induced diabetic mice were treated orally either with 30 mg/kg of F-FTS (*n* = 5) or, as a control, with carboxymethyl cellulose (CMC) vehicle **(** *n = *5). Thirteen weeks later, the mice were injected i.v. with 500 µg of 2-NBDG. Two hours after injection, muscle and liver tissues were removed and analyzed for glucose uptake as described above.

### Ras inhibition and effect on an in-vivo model of type 2 diabetes

To study the effect of F-FTS on type 2 diabetes in high fat diabetic mice, we started to treat them, at the same time as the high-fat diet was initiated, with five different daily treatments, as follows: F-FTS (20 mg/kg body weight), injected intraperitoneally (i.p.; *n* = 30); F-FTS (30 mg/kg body weight), per os (p.o.; *n* = 10); FTS (60 mg/kg body weight), p.o. (*n* = 10); CMC (control), p.o. (*n* = 10); or PBS (control), i.p. (*n* = 30). Mice were considered diabetic when two consecutive blood tests, collected from the mice's orbital sinus, yielded glucose concentrations greater than 200 mg/dl glucose. This value was obtained by all mice after 13 weeks on the diet. Prior to euthanasia of mice, fasting serum insulin levels were determined by ELISA according to the manufacture's instructions.

## Results

### Ras inhibition *in vitro* enhances glucose uptake via IkB/NF-**κ**B signaling pathway

**Figure 1:**
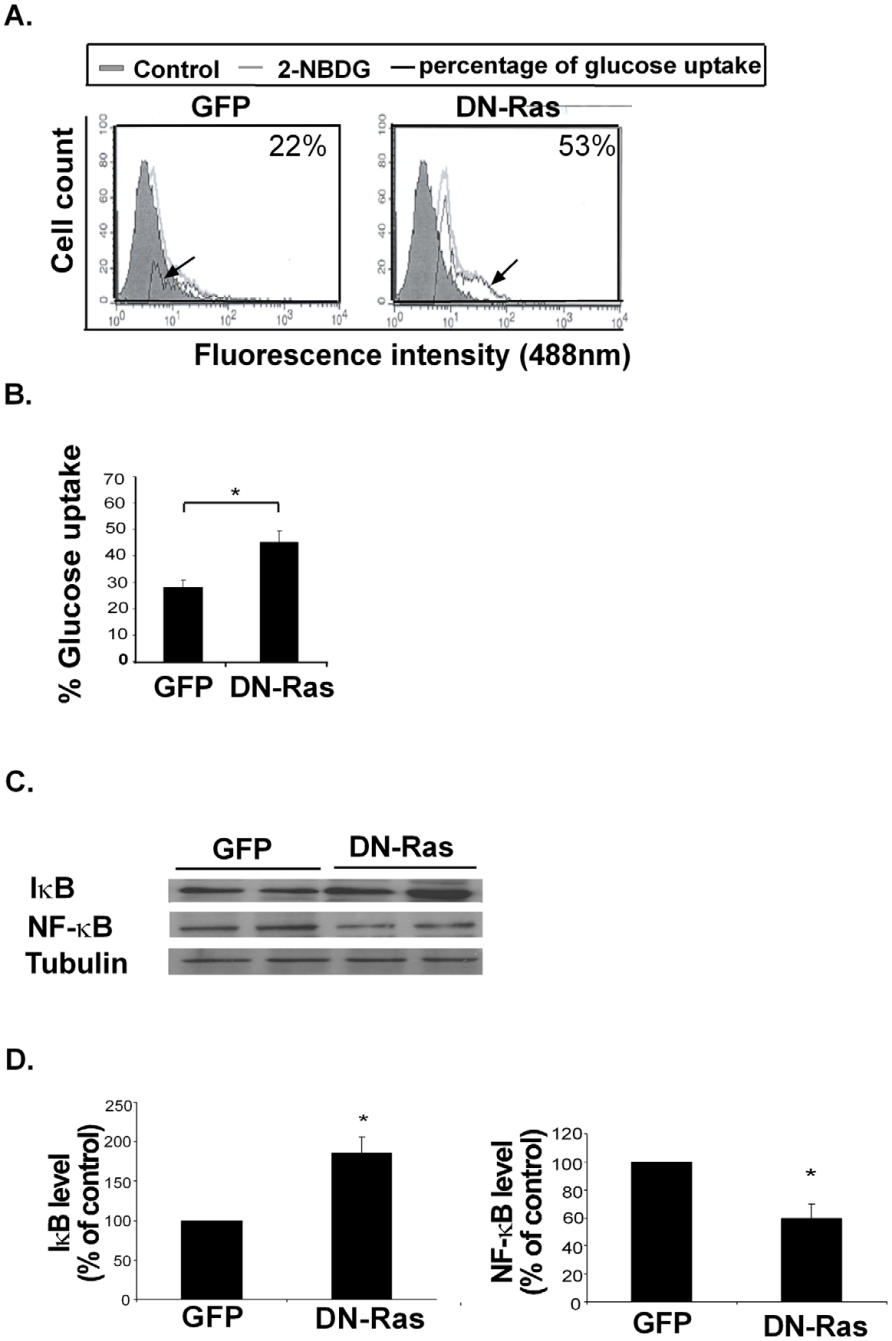
Inhibition of Ras *in vitro* by DN-Ras increases glucose uptake and alters IKB/ NF-κB expression. **A.** Insulin-resistant C2C12 myotubes were transfected with DN-Ras-GFP or GFP plasmid (pGFP) and fluorescent glucose uptake was measured by flow cytometry. Representative histograms of glucose uptake are presented (*n* = 4) **B.** Statistical analysis of the results is presented as means ± S.D. * *P*<0.05. **C.** IκB, NF-κB and tubulin expression in the DN-Ras transfected or GFP-transfected myotubes were assayed by western blotting, as described in Material and Methods. Representative blots are presented (*n* = 4). **D.** Densitometry of IκB and NF-κB expression. * *P*<0.05 compared to control.

To test the hypothesis that Ras inhibition results in increased uptake of glucose in muscle cells, we induced C2C12 cell differentiation into myotubes (see Methods) and transfected the differentiated muscle cells with DN-GFP-labeled Ras or a GFP-labeled control plasmid. We then induced insulin resistance in all cells by addition of palmitate (see Methods) and assayed their ability to absorb fluorescent glucose. In line with previous reports [35], palmitate reduced glucose uptake compared to BSA-treated control cells (not shown). The results clearly demonstrate a significant increase of 160%±10% in glucose uptake in DN-Ras transfected cells treated with palmitate as compared to palmitate-treated GFP-transfected cells (Fig. 1A and B). These results suggest that active Ras inhibition may upregulate glucose absorption.

The nuclear transcription factor NF-κB and its inhibitor IκB have been shown to participate in the induction of insulin resistance by palmitate. To study the effect of Ras inhibition on NF-κB and IκB in the insulin-resistant C2C12 cells, we assayed IκB and NF-κB in these cells following DN-Ras transfection. Compared to the GFP-transfected controls, the DN-Ras transfected cells exhibited a significant increase in their IκB expression (186%±14% increase) as opposed to a decrease in NF-κB expression (45%±10% decrease) relative to GFP-transfected cells (Fig. 1C and D).

### Inhibition of Ras by the small molecule F-FTS *in vitro* promotes Glut4 expression and induces glucose uptake in an IκB/NF-κB-dependent manner

To verify the effect of Ras inhibition on glucose uptake, we used F-FTS, a small molecule that interferes with anchorage of Ras to the membrane and hence inhibits Ras function [29], [31].

**Figure 2 pone-0021712-g002:**
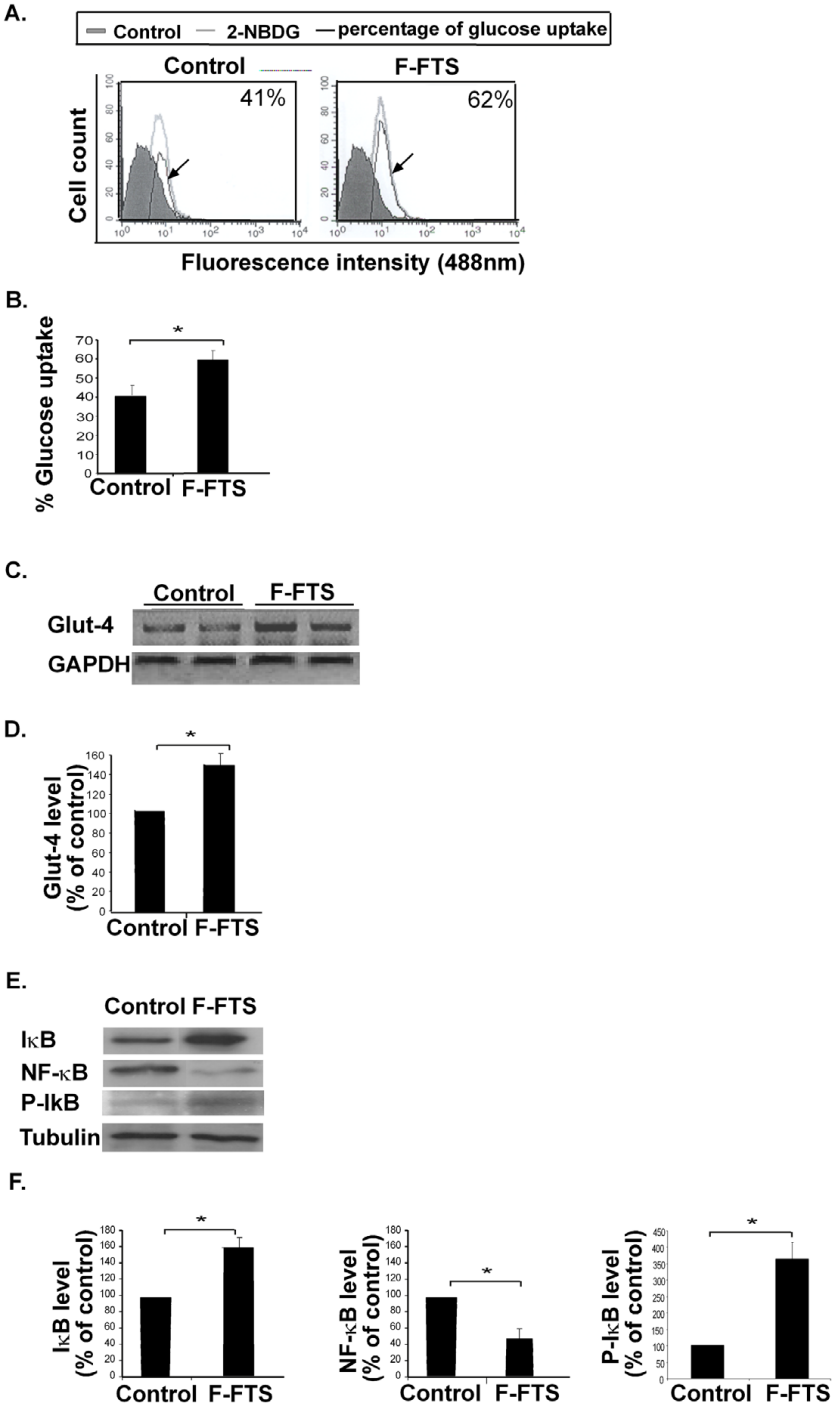
F-FTS induces glucose uptake *in vitro* and influences expression of Glut4 mRNA and of IKB/NF-κB protein. **A.** Insulin-resistant C2C12 myotubes were incubated with or without F-FTS (50 µM), and were then assayed for their ability to absorb fluorescent glucose. Representative histograms of glucose uptake are presented (*n* = 4) **B.** Statistical analysis of the results is presented as means ± S.D. * *P*<0.05. **C.** F-FTS-treated C2C12 cells were tested for Glut4 mRNA and GAPDH mRNA by RT−PCR. Representative gels are shown (*n* = 4). **D.** Densitometry of Glut4 is shown. * *P*<0.05 compared to control. **E.** IKB, NF-κB, p-IKB and tubulin were assayed by western blotting as described in Methods. Representative blots are presented (*n* = 4) **F.** Densitometry of IκB, p-IKB and NF-κB expression. * *P*<0.05 compared to control.

First, we examined whether F-FTS mimics the observed effect of DN-Ras on glucose uptake in C2C12 myotubes. Our results showed that F-FTS, like DN-Ras, induced a significant increase in glucose uptake compared to control (151%±7%, Fig. 2A and B). In line with this effect of F-FTS on glucose uptake was the finding that expression of the mRNA glucose transporter 4 (glut-4) was induced by F-FTS. Glut-4 expression in C2C12 myotubes that were rendered insulin-resistant by palmitate and then treated with F-FTS was significantly higher (146%±8%) than in C2C12 myotubes treated with palmitate alone (Fig. 2C and D).

In addition, the level of the proinflammatory transcription factor NF-κB in the insulin-resistant C2C12 cells was significantly lower (by 56%±10%) in the presence of F-FTS than in its absence (Fig. 2E and F). On the other hand, the expression of its inhibitor, IκB, was significantly higher both in its total level and in its phosphorylated form (p- IκB) by 160%±14% and 369%±35%, respectively in the presence of F-FTS compared to control (Fig. 2E and F). These findings are consistent with the results obtained by treatment with DN-Ras.

### Ras inhibition by hydrodynamic injection of DN-Ras induces glucose uptake *in vivo*

**Figure 3 pone-0021712-g003:**
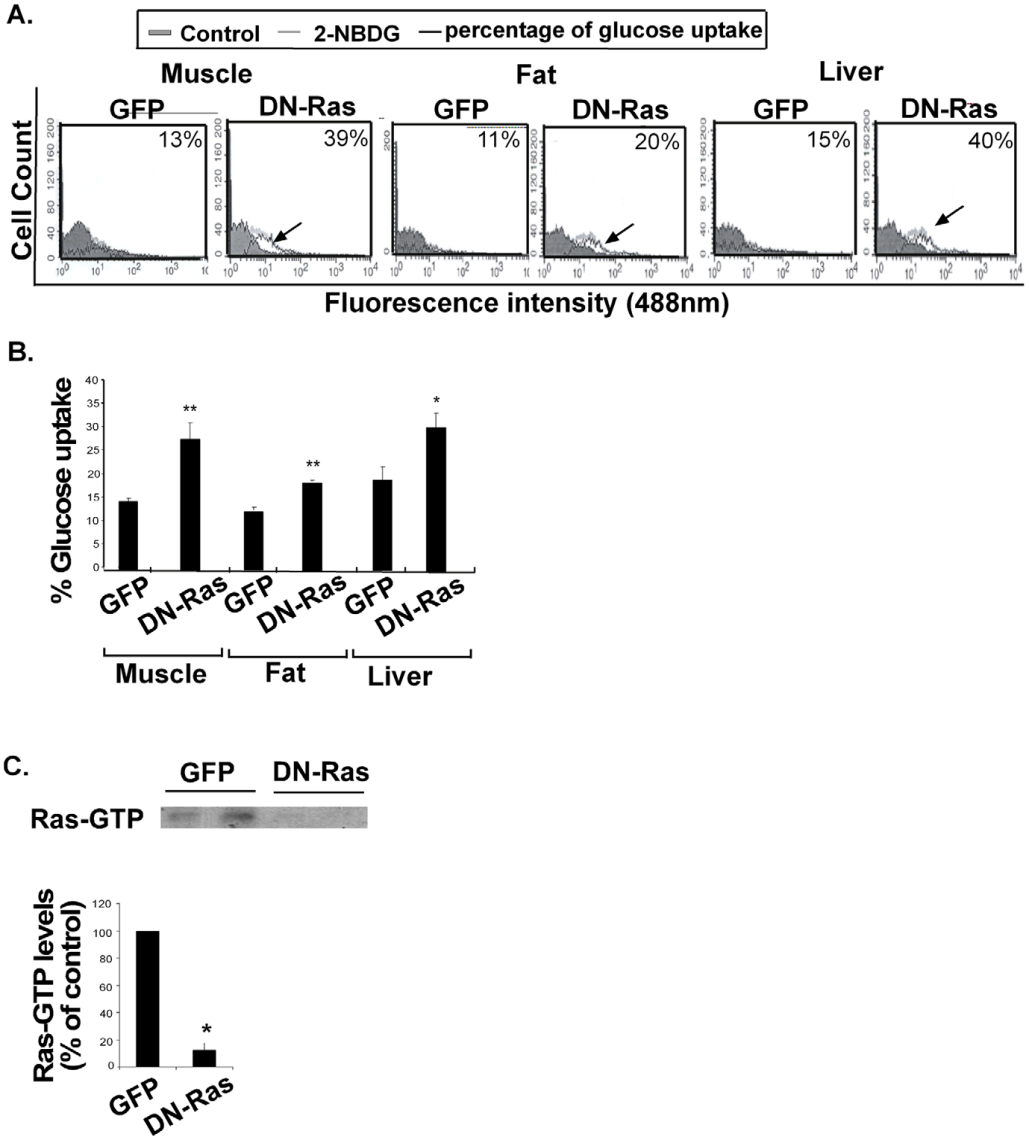
Ras inhibition *in vivo* increases muscle, fat and liver glucose uptake. **A**. HF-induced C57/Bl mice were hydrodynamically injected (i.v.) with DN-GFP-Ras or with pGFP, as described in Methods. Mice were injected with the fluorescent glucose analog 2-[*N*-(7-nitrobenz-2-oxa-1,3-diazol-4-yl)] (2-NBDG) and glucose uptake in muscle, fat and liver tissues was assayed (*n* = 8). Representative histograms of glucose uptake are presented for each tissue. **B.** Statistical analysis of the results is presented as means ± S.D. * *P*<0.05, **P<0.01. **C.** Representative gels and densitometry of Ras-GTP are shown (*n* = 4). * *P*<0.05 compared to control.

To determine whether the enhanced glucose uptake resulting from treatment with DN-Ras *in vitro* (Figs. 1 and 2) is also observed in a short-term *in vivo* model, we examined the effects of Ras inhibition on the uptake of fluorescent glucose by muscle, fat and liver tissues in mice. Hydrodynamic injection of DN-Ras into the tail veins of wild-type C57Bl/6 mice resulted in a significant increase in fluorescent glucose uptake in these tissues (an increase of 214%±10%, 150%±8%, and 157%±16% relative to the GFP-treated controls, respectively) (Fig. 3A, B).

Testing of the above tissues for Ras-GTP expression revealed a significant decrease, concomitantly with the effects on glucose uptake (Fig.3C).

### F-FTS treated mice exhibit increased glucose uptake accompanied by altered IκB/NF-κB expression

**Figure 4:**
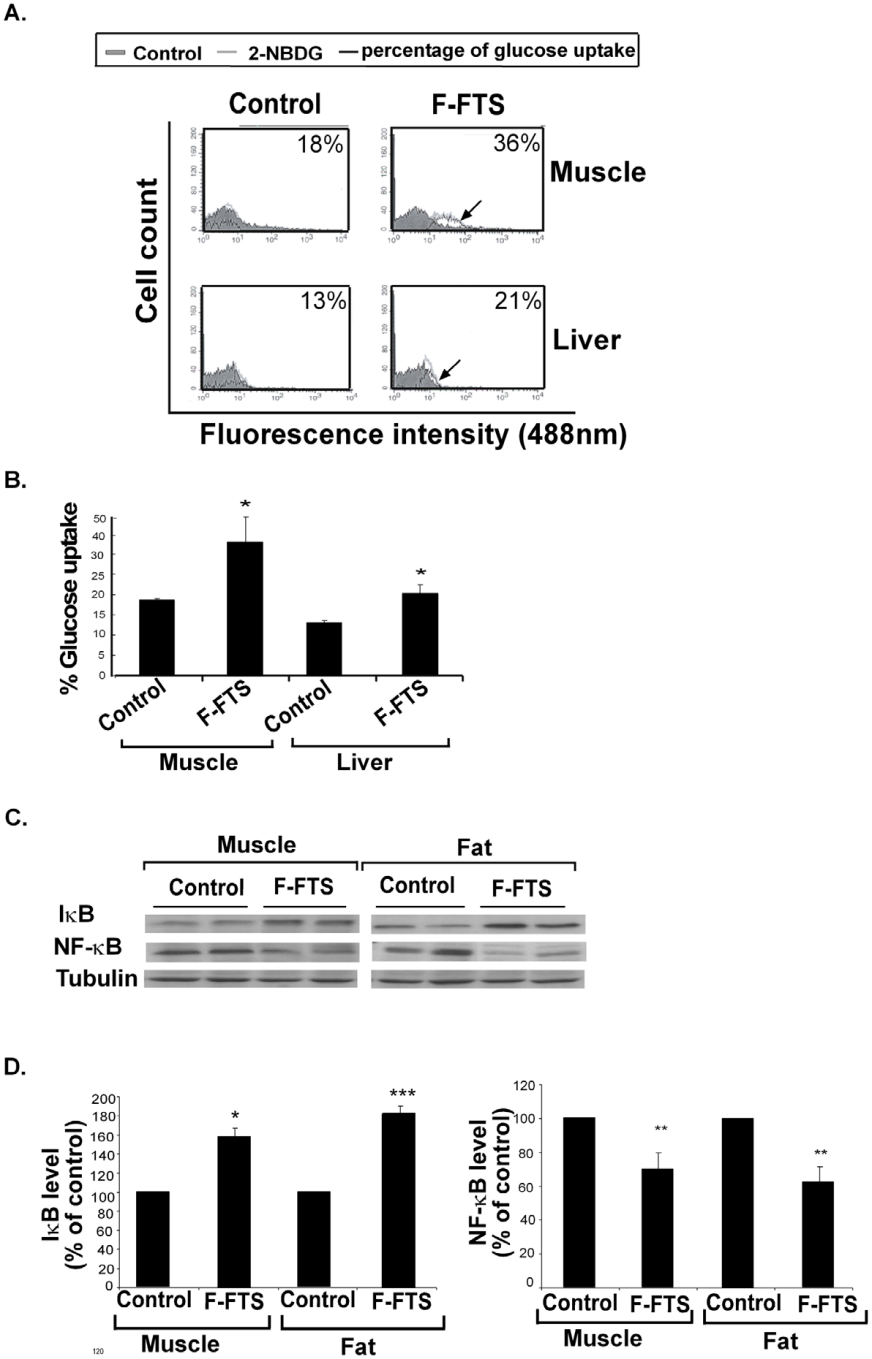
F-FTS treatment *in vivo* upregulates glucose uptake by muscle and liver tissues, accompanied by altered IκB/NF-κB expression. **A**. HF-induced C57/Bl mice treated orally with F-FTS (n = 5) or PBS (control) (n = 5) were injected i.v with 2-NBDG, and glucose uptake in their muscle and liver tissues was tested (*n* = 5). Representative histograms of glucose uptake are presented for each tissue. **B.** Statistical analysis of the results is presented as means ± S.D. * *P*<0.05. C. IκB, NF-κB and tubulin in the tissues were assayed by western blotting, as described in Methods. Representative blots are presented (*n* = 5). D. Densitometry of IκB and NF-κB expression. * *P*<0.05, **P<0.01, ***P<0.005 compared to control.

To verify the above *in vivo* findings in a relevant type 2 diabetes model, we treated 6-week-old C57Bl/6 mice fed on a high fat diet with F-FTS or, as a control, with PBS for 13 weeks, as described above, and then examined the ability of their muscle, fat and liver tissues to absorb intravenously injected fluorescent glucose. Cells obtained from the muscle and liver tissues of F-FTS-treated mice exhibited a significant increase in fluorescent glucose uptake compared to control (178±18% and 153±7%, respectively; Fig. 4A, B). No significant differences in glucose uptake were observed in the fat tissues (data not shown).

Immunoblot assays for IκB and NF-κB expression showed increased IκB expression compared to controls in muscle (by 158±10%) and fat (by 181±13%) tissues obtained from F-FTS treated mice, as opposed to a significant decrease compared to controls in NF-κB expression by 30±10% and by 38±8%, respectively (Figure 4C, D). No differences were found in the liver tissue (data not shown). Taken together, these results showed that Ras inhibition caused an increase in IκB/NF-κB-dependent glucose uptake *in vivo*.

### Ras inhibition by F-FTS attenuates type 2 diabetes *in vivo* and reduces circulating insulin levels

Having shown that Ras inhibition by F-FTS mimics the effect of DN-Ras on glucose uptake both *in vitro* and *in vivo*, we next examined the effect of long-term treatment with F-FTS or FTS on the development of hyperglycemia in an experimental model of type 2 diabetes.

**Figure 5:**
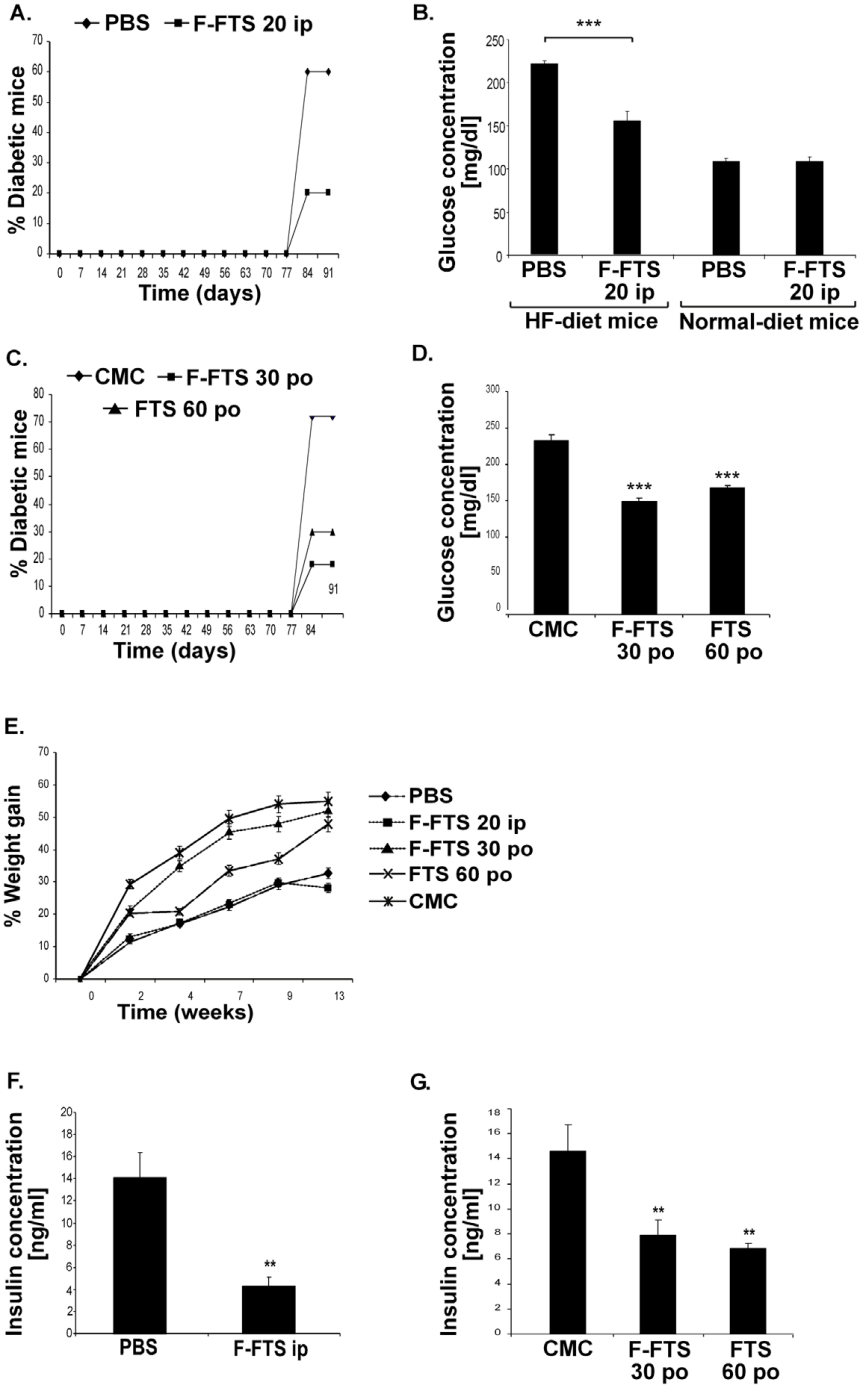
Ras inhibition in HF-induced diabetic mice reduces diabetes incidence and increases the concentration of circulating insulin. **A**. C57/Bl mice fed on a high-fat diet were treated daily with F-FTS (20 mg/kg body weight; i.p.; *n* = 30 mice per group) or PBS (*n* = 30) for 13 weeks. Kaplan-Meier plots of mean incidence of diabetes in each group. **B**. Blood glucose levels were measured as described in Methods (*n* = 10 in each group). *** *P*<0.005 compared to control. **C**. C57Bl/6 mice on a high -fat diet were treated daily with F-FTS (30 mg/kg; *n* = 10), FTS (60 mg/kg; *n* = 10) or CMC (*n* = 10) for 13 weeks. Kaplan-Meier curves record the mean incidence of diabetes in each group. **D**. Blood glucose levels were measured as described in Methods (*n* = 10 in each group). *** *P*<0.005 compared to control. **E**. All treated animals were monitored for weight gain while being fed a high-fat diet. Kaplan-Meier curves record the mean percentage of weight gain in each group. **F**, **G**. Serum insulin concentrations were measured by ELISA as described in Methods (*n* = 10 in each group). ** *P*<0.01 compared to control.

To examine how the effectiveness of the drugs was influenced by the route of administration, we treated the mice either intraperitoneally (i.p.) or per os (p.o.). While being fed from the age of 6 weeks with a high-fat diet to induce diabetes, C57Bl/6 mice were also treated i.p., daily for 13 weeks, with either 20 mg/kg of F-FTS (*n* = 30) or with PBS (*n* = 30). F-FTS treatment resulted in a significant decrease in the incidence of diabetes, with 20% of the F-FTS treated mice developing diabetes as compared to 60% in the PBS-treated group (Fig. 5A). Similar results were observed when mice were treated p.o with either 30 mg/kg F-FTS or 60 mg/kg FTS compared to CMC treatment: diabetes developed in 18% of the F-FTS-treated and in 30% of the FTS-treated mice, whereas in the CMC-treated mice 72% developed diabetes (Fig. 5C). Animals were considered diabetic when blood glucose levels exceeded 200 mg/dl (Fig 5B, D).

As expected, the high-fat diet caused a significant increase in body weight in all treated groups [36]. No significant differences in weight gain were observed between the i.p. F-FTS-treated and PBS-treated groups or between the p.o FTS-treated, F-FTS-treated and CMC-treated groups (Fig. 5E).

We also assayed circulating insulin in the different groups of mice. After i.p. treatment, the levels of circulating insulin were significantly decreased in the F-FTS-treated group (4.27±1.4 ng/ml relative to 14±4.9 ng/ml in the PBS-treated group). In the orally treated mice, insulin concentrations in the F-FTS-treated and the FTS-treated groups were also significantly reduced (7.9±1.3 ng/ml and 6.8±1.1 ng/ml, respectively, relative to 14.6±2 ng/ml in the CMC-treated group; Fig. F,G).

## Discussion

Activated Ras plays an important role in modulating a number of signaling molecules that trigger cell proliferation, differentiation, and survival [37], [38]. These observations are in line with several studies showing that Ras inhibition attenuates inflammatory responses in experimental models [25], [31], [39], [40].

Resistance to insulin, resulting in decreased glucose uptake, is a major factor contributing to the development of type 2 diabetes [41]. The mechanisms responsible for inducing resistance to insulin are not completely understood, but accumulating data point to a robust association between insulin resistance and inflammation. Obesity promotes insulin resistance by resulting in a state of chronic inflammation that involves production of proinflammatory cytokines (TNF-α, IL-6), an increase in the number of macrophages, and activation of a complex cascade of signaling events in muscle, fat and liver tissues [6], [35], [42]. Consistent with these findings, we showed here for the first time that inhibition of Ras by DN-Ras or F-FTS, promoted anti-inflammatory response in a muscle cell line and in mouse tissues.

This study is the first to show a clear association between Ras signaling and insulin resistance in muscle, fat and liver. We found that inhibition of Ras activation by transfection with DN-Ras or by treatment with the small-molecule Ras inhibitor F-FTS induced glucose uptake *in vitro*, indicating higher insulin sensitivity.

In addition, we demonstrated that inhibition of Ras *in vivo* by hydrodynamic injection of DN-Ras or by daily treatment with F-FTS in an experimental murine model of HF-induced diabetes resulted in similar findings of increased uptake of fluorescently labeled glucose by muscle, fat and liver tissues.

**Figure 6:**
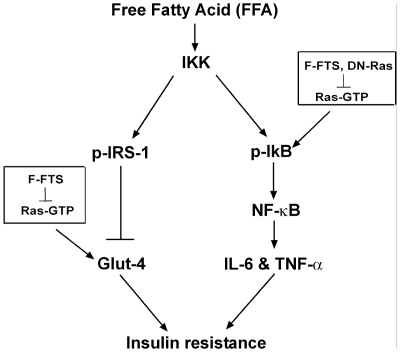
Proposed mechanism explaining the effect of Ras on insulin sensitivity. Free fatty acids (FFAs) lead to activation of IKK, the inhibitor of IκB kinase. IKK affects insulin sensitivity and glucose uptake via two distinct pathways. First, IKK phosphorylates insulin receptor substrate 1 (IRS-1), resulting in inactivation of insulin signaling through attenuated transcription of glucose transporter 4 (Glut4). Ras inhibition by F-FTS demonstrates enhanced Glut4 transcription, hence also heightened glucose uptake. Second, IKK phosphorylates the inhibitor of κB (IκB), causing it to become detached from nuclear factor κB (NF-κB). NF-κB enters the nucleus and induces transcription of proinflammatory cytokines such as IL-6 and TNF-α. These cytokines leads to deterioration of insulin resistance. Ras inhibition by DN-Ras or by F-FTS augments IκB expression, thereby attenuating the proinflammatory response and enhancing insulin sensitivity and glucose uptake.

To characterize the signaling pathway by which Ras inhibition promotes insulin sensitivity, we studied the expression of key regulators known to participate in insulin-signaling pathways. For example, activation of the IκB/NF-κB cascade activates a widespread proinflammatory program. IκB kinase (IKK) phosphorylates certain serine residues on insulin receptor kinase 1 (IRS-1), leading to impairment of insulin signal transduction. In addition, the IKK signaling pathway is upregulated and activated, both in insulin-resistant humans and in rodent skeletal muscles [43]. Increased expression of IKK results in inhibition of IκB and activation of NF-κB ; the latter subsequently transcriptionally activates a set of inflammatory pathway genes that induce resistance to insulin (see scheme, Fig. 6)[44].

Based on the above knowledge, we sought to explore the influence of Ras inhibition on the IκB/NF-κB cascade in a conventional model of insulin resistance. We found that Ras inhibition led to an increase in IκB, which inhibited the expression of NF-κB both *in vitro* and *in vivo* (Figs. 1–4). The improvement in glucose uptake in liver tissue of the F-FTS treated animals was not correlated with increased expression of IκB (Fig 4). This finding could result from the long period (13 weeks) of treatment that may influence the duration of the increased IκB expression. Overall, the observation that insulin resistance was attenuated by Ras inhibition in association with regulation of IκB and NF-κB provides a possible link between Ras, inflammation, and negative regulation of insulin signaling.

The most downstream factor in the insulin cascade is Glut4, an essential transporter responsible for translocation of insulin-regulated glucose into the cell [45]. We therefore examined the effect of Ras inhibition on Glut4 mRNA levels in insulin-resistant C2C12 myotubes treated with F-FTS. We found an increase in Glut4 mRNA levels after F-FTS treatment. These results suggested that the higher sensitivity to insulin was attributable to Ras inhibition, which may be related to the increase in expression of Glut4 transporter in the plasma membrane and the subsequent potentiated influx of glucose into the cell (see scheme, Figure 6). Taken together, our results suggest dual affects of Ras on insulin sensitivity and glucose uptake via two distinct pathways (Figure 6).

Previous studies have shown that both FTS and the small synthetic molecule F-FTS act primarily by inhibiting active Ras proteins and are mimicked by dominant negative Ras [30], [46]. Therefore, mice fed a high-fat diet and concomitantly treated with F-FTS may serve as an appropriate *in vivo* model for examining the effect of Ras inhibition on an experimental model of type 2 diabetes. Our results showed that treatment with either FTS or F-FTS significantly attenuated the incidence of hyperglycemia in this model. The potential contribution of Ras-mediated insulin sensitization in this *in vivo* model is supported by the finding that circulating insulin levels were decreased in the FTS-treated mice (Fig. 5). The observed decrease of insulin level most likely resulted from the increased uptake of glucose into the tissues but could also be caused by a direct effect on the pancreas. Further studies should be performed to clarify the cause of the decrease in serum insulin levels.

Taken together, the results of this study showed that inhibition of Ras signaling enhances both insulin sensitivity and glucose uptake *in vitro* and *in vivo*. These observations were corroborated by the beneficial effects of Ras inhibition that resulted in attenuation of hyperglycemia in a conventional type 2 diabetes model. It should be noted, however, that Ras inhibition may modify inflammatory responses in other tissues as well. These findings pave the way for a novel approach to the potential treatment of insulin resistance and type 2 diabetes.
